# WJ-39, an Aldose Reductase Inhibitor, Ameliorates Renal Lesions in Diabetic Nephropathy by Activating Nrf2 Signaling

**DOI:** 10.1155/2020/7950457

**Published:** 2020-05-30

**Authors:** Xiaoyu Zhou, Zheng Liu, Ke Ying, Huimin Wang, Peng Liu, Xuefei Ji, Tianyan Chi, Libo Zou, Shaojie Wang, Zhonggui He

**Affiliations:** ^1^Department of Pharmacology, Shenyang Pharmaceutical University, 103 Wenhua Road, Shenhe District, Shenyang Liaoning 110016, China; ^2^Zhejiang CONBA Pharmaceutical Co., Ltd., Hangzhou 310052, China; ^3^Department of Pharmacochemistry, Shenyang Pharmaceutical University, 103 Wenhua Road, Shenhe District, Shenyang Liaoning 110016, China; ^4^Department of Pharmaceutics, Shenyang Pharmaceutical University, 103 Wenhua Road, Shenhe District, Shenyang Liaoning 110016, China

## Abstract

Diabetic nephropathy (DN) is a chronic diabetic microvascular complication. Hyperactivity of the polyol pathway is involved in the pathogenesis of DN. Aldose reductase (AR), the rate-limiting enzyme of the polyol pathway, is expected to be an effective target in the treatment of DN. WJ-39 is a novel inhibitor of AR. The present study aimed at exploring the effects of WJ-39 in DN. DN was induced in rats by injecting 30 mg/kg streptozotocin (STZ). After 14 weeks, WJ-39 (10, 20, and 40 mg/kg) was intragastrically administered to the rats for 12 weeks. Treatment with WJ-39 significantly inhibited AR activation and ameliorated renal dysfunction and fibrosis in DN rats. WJ-39 reduced oxidative stress in the kidneys of DN rats by activating the nuclear factor erythroid 2-related factor 2 (Nrf2) pathway. WJ-39 suppressed the activation of the nuclear factor-kappa B (NF-*κ*B) pathway and the nucleotide-binding and oligomerization domain-like receptor family pyrin domain-containing 3 (NLRP3) inflammasome to reduce the secretion of inflammatory factors. Rat mesangial cells (RMCs) were cultured under hyperglycemic conditions. WJ-39 abrogated the high glucose- (HG-) induced, excessive production of reactive oxygen species (ROS) and inflammatory factors. However, transfection with Nrf2 small interfering RNA abolished the effects of WJ-39. WJ-39 also blocked the transforming growth factor-*β*1/Smad pathway to reduce the production of glomerular extracellular matrix proteins, ultimately reducing fibrogenesis in DN. Our results show that WJ-39 ameliorated renal injury in DN rats, and its effects on oxidative stress and inflammation were associated with the activation of Nrf2 signaling. Thus, WJ-39 and its mechanism of amelioration of renal lesions in DN rats by reducing renal inflammation, oxidative stress, and fibrosis injury could be an effective strategy for the treatment of DN.

## 1. Introduction

In China, there are an estimated 110 million people with diabetes, accounting for approximately 11% of the population [1]. Approximately 30–40% of diabetes patients develop diabetic nephropathy (DN) [2], a common complication of both types I and II diabetes. In addition, DN is the main cause of end-stage renal disease in almost 75% of type I diabetic patients and 20% of type II diabetic patients [3]. The etiology of DN is multifactorial and obscure. Oxidative stress is an initiating factor in DN, fibrosis is generally the terminal outcome of kidney disease, and inflammation seems to be pivotal in the initiation and progression of fibrosis [4]. Sustained hyperglycemia in cells leads to oxidative stress through the generation of free radicals and disrupts the intracellular antioxidative mechanism. The nuclear factor erythroid 2-related factor 2 (Nrf2) pathway is an important defense system against oxidative stress and is responsible for the transcription of cytoprotective genes such as the one encoding the antioxidative protein, heme oxygenase-1 (HO-1) [5]. However, Nrf2 activity is disrupted, and the expression of its target gene products is also reduced in DN. Abundant evidence suggests that there is cross talk between the Nrf2 and nuclear factor-kappa B (NF-*κ*B) pathways, which activates the transcription of genes that produce inflammatory cytokines [6, 7]. Activation of the Nrf2 pathway inhibits NF-*κ*B activity [6]. Therefore, diminished activation of Nrf2 in DN contributes to oxidative stress and inflammation, further amplifying the damaging effect on the kidney. The nucleotide-binding and oligomerization domain-like receptor family pyrin domain-containing 3 (NLRP3) inflammasome is another cause of renal inflammatory injury in DN, and it converts the proinflammatory cytokines, interleukin- (IL-) 1*β* and IL-18, to their active forms in renal cells [8, 9]. Overactivation of the NLRP3 inflammasome can be inhibited by activating the Nrf2 pathway [10, 11]. Various injury stimuli eventually lead to renal fibrosis. Transforming growth factor-*β*1 (TGF-*β*1) is a profibrotic cytokine that is linked to glomerular pathobiology in renal disease [12, 13]. In DN, TGF-*β*1 stimulates the synthesis and excessive deposition of extracellular matrix (ECM) components via different signaling pathways (e.g., Smad), which lead to the thickening of the glomerular basement membrane and mesangial expansion [14, 15]. These pathways are activated and participate in the process of DN through close cross talk and mutual reinforcement.

Regardless of how complex the mechanism is, dysregulated glucose homeostasis in renal cells is recognized as the key pathogenic factor in DN [4]. In DN, excess glucose enters susceptible renal cells, creating hyperglycemic conditions that stimulate more than 30% of glucose metabolism through the polyol pathway, making it an important pathway that results in oxidative stress and inflammatory reactions. Aldose reductase (AR) is the first and rate-limiting step in the polyol pathway [16–18]. Increasing evidence from preclinical and clinical studies indicates that suppressing AR activity is beneficial for the treatment of DN [19]; aldose reductase inhibitors (ARIs) may arrest or even reverse the progression of DN. Li et al. and Jing et al. had reported the synthesis and pharmacokinetic study of the ARI, WJ-38 (darirestat) [20, 21]; WJ-39 is a potassium salt of WJ-38. This study investigated the mechanism of WJ-39 in DN by using a streptozotocin- (STZ-) induced DN rat model and rat mesangial cells (RMCs) cultured under high glucose (HG) conditions.

## 2. Materials and Methods

### 2.1. Animal Experiments

Male Sprague-Dawley (SD) rats (adult, 180–200 g weight) were purchased from Beijing Huafukang Bioscience Co., Ltd., Beijing, China. During the experiments, the animals were housed in a controlled environment with free access to food and water. The rats were exposed to a constant temperature of 25 ± 2°C and humidity of 50 ± 10%, with alternating 12 h light and dark cycles. All animal procedures were approved and performed in accordance with the legislation of the People's Republic of China on the use and care of laboratory animals and the guidelines established by the Institute for Experimental Animals at Shenyang Pharmaceutical University (permit number: SYPU-IACUC-S2017-04.26-201).

**Figure 1:**
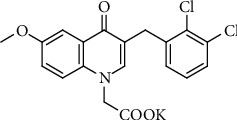
Structure of WJ-39.

Induction of diabetes was carried out in the experimental animals, after overnight fasting, with a single intravenous tail injection of STZ (30 mg/kg, Sigma-Aldrich, St. Louis, MO, USA, prepared in 0.1 M citrate buffer, pH = 4.5). Additionally, 15 rats were injected with citrate buffer alone and served as controls. Fasting blood glucose levels were detected at 72 h after STZ injection. The diabetic model was considered to be successfully established when the fasting blood glucose level was ≥16.7 mmol/L (300 mg/dL) and was further evaluated by the oral glucose tolerance test (OGTT) one week after STZ injection. The rats were maintained on a standard rodent diet with water ad libitum. Ten weeks after STZ injection, urine was collected for measurement of ACR (albumin-to-creatinine ratio) by placing the rats in metabolic cages for 24 h once a month. After a significant difference in ACR had developed between the diabetic rats and normal rats (14 weeks after STZ injection), the DN rats were divided into six groups (*n* = 18–24 each group): DN rats without treatment (STZ-induced DN rats), DN rats treated with WJ-39 (10, 20, and 40 mg/kg + 0.5%sodium carboxymethylcellulose (SCMC)), DN rats treated with irbesartan (30 mg/kg + 0.5%SCMC), and DN rats treated with epalrestat (15 mg/kg + 0.5%SCMC). The doses of WJ-39, irbesartan (a first-line drug for treatment of DN) and epalrestat (an ARI) were based on reports [22–24] and our previous study. The agents were administered intragastrically via oral gavage once a day for 12 weeks. Rats in the control group (*n* = 15) and DN rats without treatment received equal volumes of 0.5% SCMC. WJ-39 (purity: 99.97%, Figure 1) was provided by Kangya of Ningxia Pharmaceutical Co., Ltd, Ningxia, China. The rats were anesthetized with an intraperitoneal injection of chloral hydrate (300 mg/kg) and sacrificed, following which renal cortex tissues were harvested for subsequent experiments.

### 2.2. Albumin-to-Creatinine Ratio (ACR) and Creatinine Clearance Rate (Ccr)

ACR and Ccr were measured by using assay kits according to the manufacturer's protocols (Tianjin Anoric Bio-Technology, Tianjin, China).

### 2.3. Superoxide Dismutase (SOD), Malondialdehyde (MDA), Catalase (CAT), Reduced/Oxidized Glutathione (GSH/GSSG), Oxidized/Reduced Form of Nicotinamide-Adenine Dinucleotide (NAD^+^/NADH), and Aldose Reductase (AR)

MDA levels, activities of SOD and CAT, ratios of GSH/GSSG and NAD^+^/NADH (Nanjing Jiancheng Biology Engineering Institute, Nanjing, China), and AR activity (HepengBio, Shanghai, China) in rat renal cortex tissues were measured by using assay kits according to the manufacturers' protocols.

### 2.4. Histology and Immunostaining

Kidney tissue sections were fixed in 4% paraformaldehyde and subsequently processed for paraffin sectioning. The renal tissue sections (5 *μ*m thickness) were stained with periodic acid-Schiff (PAS) and Masson's trichrome stains. Images were acquired by an inverted microscopy (Olympus IX71, Tokyo, Japan). Mesangial matrix index (area of PAS staining/total area) and percentage of fibrosis (area of Masson's trichrome staining/total area) were measured by using the Image-Pro Plus software (Media Cybernetics, Rockville, MD, USA).

### 2.5. Immunofluorescence Staining

The renal tissue sections were deparaffinized and rehydrated in an alcohol gradient and xylene and then boiled in citrate buffer for 20 min for antigen retrieval. After blocking with 5% goat serum for 30 min, the sections were incubated with primary antibodies: anti-fibronectin (FN, ab2413, Abcam, Cambridge, UK) and anti-collagen-IV (col-IV, ab6586, Abcam) overnight at 4°C. After washing with phosphate-buffered saline (PBS), the sections were incubated with secondary antibodies (Alexa Fluor 594-conjugated goat anti-rabbit, Proteintech, Wuhan, China) for 30 min at 37°C in the dark. The images were captured by confocal laser-scanning microscopy (Nikon C2 Plus, Tokyo, Japan). The analysis of the overlay was performed by using ImageJ (National Institutes of Health, Bethesda, MA, USA).

### 2.6. Transmission Electron Microscopy (TEM)

Renal cortices were minced into pieces (1 mm^3^) and fixed in 2.5% glutaraldehyde in PBS (0.1 M, pH 7.4). The tissue sections were washed with PBS and then fixed in 1% osmic acid. After washing with water, the samples were dehydrated using graded alcohol and anhydrous acetone. The samples were oriented longitudinally and embedded in Epon 812. Ultrathin sections (70 nm) were cut and stained with uranyl acetate and lead citrate and then examined at 80 kV under a transmission electron microscope (JEM-1200EX, JEOL, Tokyo, Japan).

### 2.7. Cell Culture and Treatments

Rat mesangial cells (RMCs, Chinese Academy of Medical Sciences Cell Center, Beijing, China) were cultured in Dulbecco's modified Eagle's medium (DMEM, HyClone, Logan, Utah, USA) containing 5.6 mmol/L glucose and 10% fetal bovine serum (FBS, CLARK, Richmond, VA, USA) at 37°C and in a 5% CO_2_ atmosphere. The cells were divided into 7 groups: (1) normal glucose (NG): 5.6 mmol/L glucose; (2) high glucose (HG): 30 mmol/L glucose; (3) various concentrations of WJ-39 (1, 10, and 100 *μ*M) + HG; (4) irbesartan (10 *μ*M) + HG; and (5) epalrestat (10 *μ*M) + HG. The cells were simultaneously treated when exposed to HG.

### 2.8. 3-(4,5-Dimethylthiazol-2-yl)-2,5-diphenyltetrazolium Bromide (MTT) Assay to Assess Cell Viability

RMCs were seeded in 96-well plates at a density of 1 × 10^4^/mL and then treated with WJ-39 (1, 10, and 100 *μ*M), irbesartan (10 *μ*M), and epalrestat (10 *μ*M) with or without HG for 24, 48, and 72 h. Then, 15 *μ*L MTT (5 mg/mL) was added to each well and incubated for 4 h at 37°C. The cells were treated with 150 *μ*L dimethyl sulfoxide, and absorbance was measured at 490 nm by using a microplate reader (Thermo Scientific-Varioskan Flash, Waltham, MA, USA).

### 2.9. Cell Counting Kit-8 (CCK-8) Assay to Assess Cell Viability

RMCs were seeded in 96-well plates at a density of 1 × 10^4^/mL and then treated with WJ-39 (1, 10, and 100 *μ*M), irbesartan (10 *μ*M), and epalrestat (10 *μ*M) with NG for 48 h. Then, 10 *μ*L of CCK-8 was added to each well, and the cells were incubated for 1 h. Absorbance was measured at 450 nm by using a microplate reader.

### 2.10. Nrf2 Small Interfering RNA (siRNA) Transfection

RMCs were seeded in 6-well culture plates and transfected with Nrf2 siRNA using Lipofectamine 2000 transfection reagent (Invitrogen, Carlsbad, CA, USA) after 24 h. RMCs were incubated with Nrf2 siRNA (Santa Cruz, Dallas, Texas, USA) and control scrambled siRNA (Ambion, Austin, Texas, USA) in serum-free Opti-MEM medium (Thermo Fisher, Waltham, MA, USA) for 4 h, which was then replaced with 10% FBS-containing DMEM for various 48 h treatments.

### 2.11. Detection of Intracellular Reactive Oxygen Species (ROS)

Intracellular ROS levels were measured by using 2′,7′-dichlorofluorescein diacetate (DCFDA) (Nanjing Jiancheng Biology Engineering Institute, Nanjing, China) according to the manufacturer's protocol. The cells were incubated with 20 *μ*M dichloro-dihydro-fluorescein diacetate (DCFH-DA) for 45 min at 37°C and then washed two times with PBS. The cells were collected and analyzed by using a microplate reader (excitation/emission: 500/525 nm).

### 2.12. Western Blotting

Nuclear and cytoplasmic proteins were extracted using a nuclear protein and cytoplasmic protein extraction kit (Beyotime, Shanghai, China). Total proteins were extracted with cell lysis radioimmunoprecipitation assay buffer (CWBIO, Beijing, China). Extracts of all samples containing 30 *μ*g protein were separated by electrophoresis on 10–12% sodium dodecyl sulfate polyacrylamide gels and electrotransferred onto 0.45 *μ*m polyvinyl difluoride membranes (Millipore, Bedford, MA, USA). The membranes were blocked with 5% skimmed milk in PBS for 2 h at room temperature and incubated with primary antibodies overnight at 4°C (Table 1). After washing 3 times with PBS, the membranes were incubated with appropriate concentrations of secondary antibodies (Thermo Scientific, Waltham, MA, USA) at room temperature for 2 h. The immunoreactive bands were visualized with an enhanced chemiluminescence kit (Advansta, Menlo Park, CA, USA), quantified by densitometry by using a molecular imager system (Bio-Rad, Hercules, CA, USA), and analyzed by using Image-Pro Plus.

### 2.13. Statistical Analysis

Results are expressed as the mean ± standard error of the mean (SEM). The data were analyzed using SPSS 17.0 (IBM, Armonk, NY, USA). Differences among groups were analyzed for statistical significance using one-way analysis of variance, followed by Fisher's least significant difference multiple comparisons test with homogeneity of variance or Dunnett's T3 test with heterogeneity of variance. The differences in OGTT between SD rats and STZ-induced diabetic rats and the differences in ACR and Ccr between two groups, before and after treatment, were analyzed for statistical significance using *t*-test. A *p* value < 0.05 was considered statistically significant.

## 3. Results

### 3.1. WJ-39 Inhibited the Activity of AR and Ameliorated Renal Dysfunction and Fibrosis in STZ-Induced DN Rats

**Figure 2:**
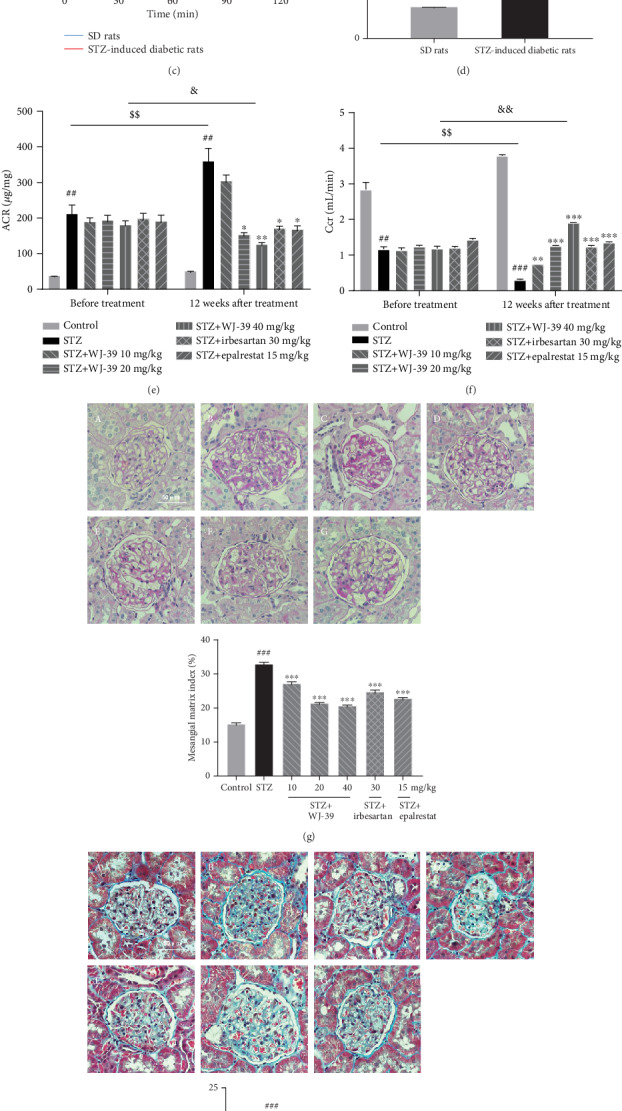
WJ-39 inhibited the activity of aldose reductase (AR) and ameliorated renal dysfunction and fibrosis in streptozotocin- (STZ-) induced diabetic nephropathy (DN) rats. (a) AR activity in renal cortex tissues was detected by using a biochemical chromatometry kit. (b) AR protein levels in renal cortex tissues were detected by western blotting and quantified. (c) Blood glucose levels and (d) area under the curve (AUC) of SD rats (*n* = 15) and STZ-induced diabetic rats (*n* = 124) in the oral glucose tolerance test (OGTT) were detected one week after STZ injection. (e) Urine albumin-to-creatinine ratio (ACR) and (f) creatinine clearance rate (Ccr) were measured before and after WJ-39 treatment (14 weeks and 26 weeks after STZ administration, respectively). (g) Representative images (400×) and mesangial matrix index of periodic acid-Schiff (PAS) staining of DN rat kidneys with different treatments (scale bar = 50 *μ*m, *n* = 33–56). (h) Representative images (400×) and percentage of fibrosis of Masson's trichrome staining of DN rat kidneys with different treatments (scale bar = 50 *μ*m, *n* = 39–60). (i) Representative images showing the changes in glomerular basement membrane thickening and podocytic processes are marked with arrows (scale bar = 2 *μ*m). A: control; B: DN rats; C, D, and E: DN rats treated with WJ-39 (10, 20, and 40 mg/kg); F: DN rats treated with irbesartan (30 mg/kg); G: DN rats treated with epalrestat (15 mg/kg). Data are represented as the mean ± standard error of the mean (SEM), *n* = 6–10. ^##^*p* < 0.01 and ^###^*p* < 0.001 vs. the control group; ^∗^*p* < 0.05, ^∗∗^*p* < 0.01, and ^∗∗∗^*p* < 0.001 vs. the STZ group; ^$$^*p* < 0.01 vs. the STZ group at 14 weeks; ^&^*p* < 0.05 and ^&&^*p* < 0.01 vs. the group (STZ+WJ-39 40 mg/kg) before treatment.

First, we investigated the effects of WJ-39 on AR activity and protein expression. AR activity and protein levels were significantly increased in STZ-induced DN rat renal tissues. WJ-39 significantly inhibited AR activity; however, it did not affect AR protein expression. Epalrestat reduced both activity and protein expression of AR (Figures 2(a) and 2(b)). One week after STZ administration, an OGTT revealed that the blood glucose levels and area under the curve (AUC) of the STZ-induced rats were significantly increased (Figures 2(c) and 2(d)), indicating that the rats had become diabetic. Fourteen weeks after STZ administration, ACR was significantly increased, whereas Ccr was significantly decreased in diabetic rats (Figures 2(e) and 2(f)), indicating that renal function was impaired in diabetic rats, and that the rats had developed DN. The DN rats were then treated with WJ-39 for 12 weeks. Twenty-six weeks after STZ administration, ACR of DN rats was significantly increased, and Ccr was significantly reduced compared to the values of DN rats 14 weeks after STZ administration, confirming the development of DN in these STZ-induced rats. Treatment with WJ-39 (40 mg/kg) for 12 weeks significantly reduced the ACR of DN rats compared to the ACR of DN rats before WJ-39 treatment (Figure 2(e)). WJ-39 also significantly reversed the decrease in Ccr (Figure 2(f)), demonstrating that WJ-39 could reverse the progression of DN. However, WJ-39 did not affect the blood glucose levels of DN rats (unpublished data). Renal lesions and fibrosis were observed in the PAS- and Masson's trichrome-stained and TEM images of DN rat kidneys. We found that both mesangial matrix index and percentage fibrosis were considerably lower in DN rats treated with WJ-39 (Figures 2(g)–2(i)).

### 3.2. WJ-39 Treatment Attenuated Renal Oxidative Stress by Activating the Nrf2 Pathway in DN Rats

**Figure 3:**
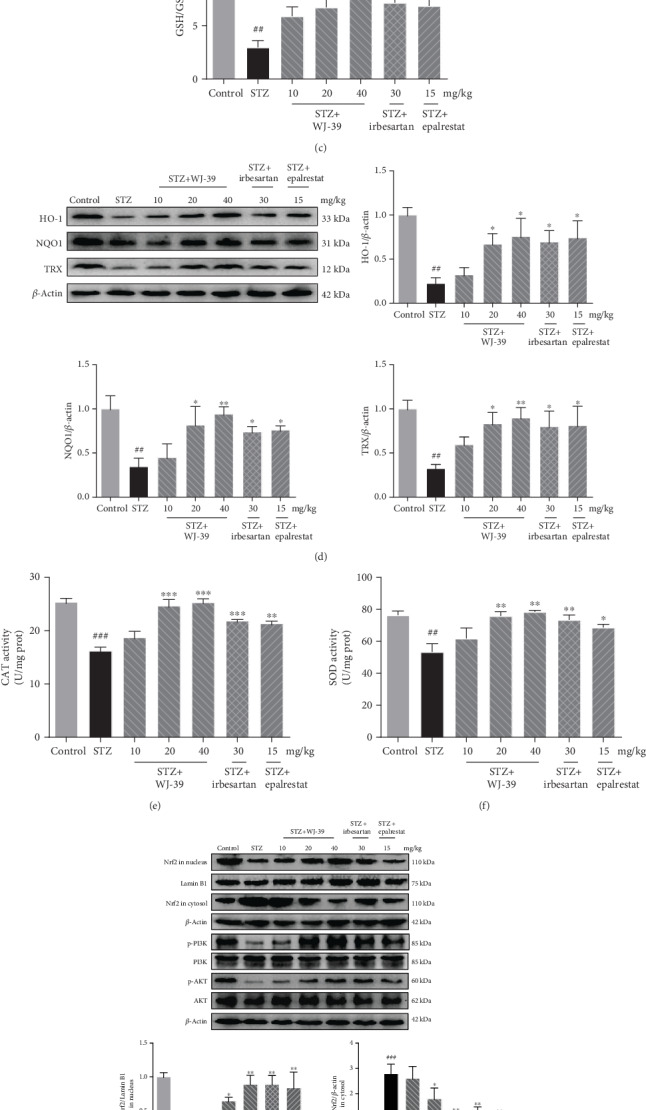
WJ-39 treatment attenuated renal oxidative stress by activating the nuclear factor erythroid 2-related factor 2 (Nrf2) pathway in diabetic nephropathy (DN) rats. The concentration of (a) malondialdehyde (MDA) and the ratios of (b) oxidized/reduced form of nicotinamide-adenine dinucleotide (NAD^+^/NADH) and (c) reduced/oxidized glutathione (GSH/GSSG) in renal cortex tissues were detected by using biochemical chromatometry kits. (d) Protein levels of heme oxygenase-1 (HO-1), NAD(P)H dehydrogenase [quinone] 1 (NQO1), and thioredoxin (TRX) in renal cortex tissues were detected by western blotting and quantified. The activities of (e) catalase (CAT) and (f) superoxide dismutase (SOD) in renal cortex tissues were detected by using biochemical chromatometry kits. (g) Protein levels of Nrf2 in the nucleus and cytosol and ratios of phosphorylated protein/total protein (p-PI3K/PI3K and p-AKT/AKT) in renal cortex tissues were detected by western blotting and quantified. Data are represented as the mean ± standard error of the mean (SEM), *n* = 6. ^##^*p* < 0.01 and ^###^*p* < 0.001 vs. the control group; ^∗^*p* < 0.05, ^∗∗^*p* < 0.01, and ^∗∗∗^*p* < 0.001 vs. the STZ group. PI3K, phosphatidylinositol 3-kinase; AKT, protein kinase B.

In DN, activation of the polyol pathway causes ROS production, disrupting the redox balance and leading to oxidative stress. Given the inhibitory effect of WJ-39 on AR activity, we hypothesized that oxidative stress in DN would be mitigated by WJ-39 treatment. Indeed, we observed that treatment with WJ-39 significantly abrogated the STZ-induced increase in MDA levels (Figure 3(a)). Treatment with WJ-39 also increased the ratios of GSH/GSSG and NAD^+^/NADH, which had decreased in STZ-induced DN rat renal tissues (Figures 3(b) and 3(c)). Endogenous antioxidant enzymes are regarded as the first line of defense against oxidative stress. The activities of CAT and SOD and the expression of HO-1, NQO1, and TRX was increased after WJ-39 treatment (Figures 3(d)–3(f)). Nrf2 is a crucial regulator of the antioxidant enzymes mentioned above. The nuclear levels of Nrf2 protein were decreased in DN renal tissues and were increased after WJ-39 treatment (Figure 3(g)). The phosphatidylinositol 3-kinase (PI3K)/protein kinase B (AKT) pathway is a crucial regulator of Nrf2 signaling to protect cells against oxidative stress. Treatment with WJ-39 significantly abrogated the STZ-induced decrease in the phosphorylation of PI3K/AKT in renal tissues (Figure 3(g)). These data suggest that the antioxidant effect of WJ-39 is linked to the PI3K/AKT/Nrf2 pathway.

### 3.3. WJ-39 Alleviated Oxidative Stress in RMCs Cultured under High Glucose Conditions

**Figure 4:**
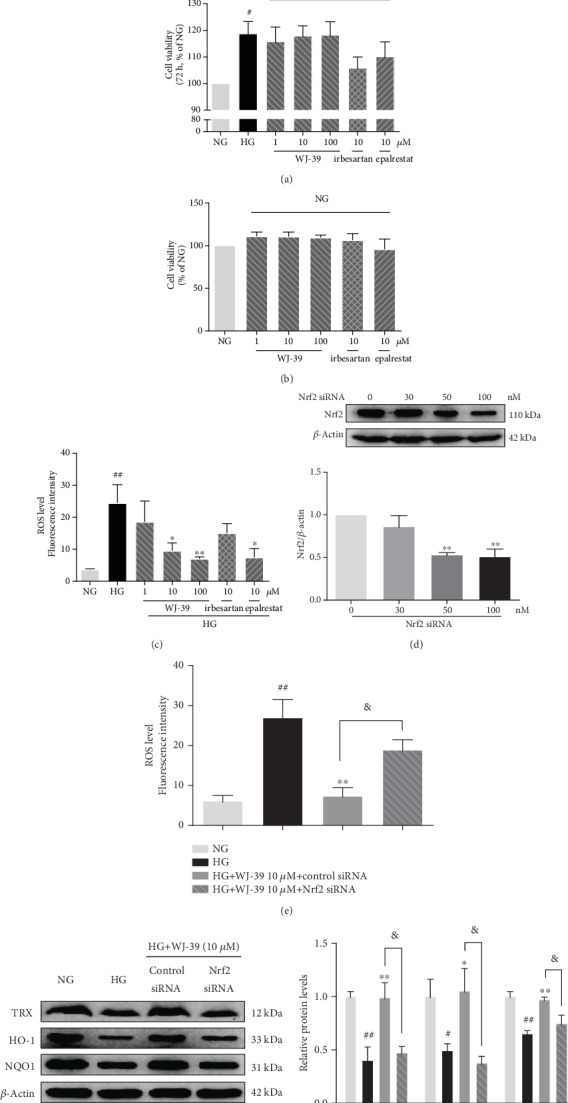
WJ-39 alleviated oxidative stress in rat mesangial cells (RMCs) cultured under high glucose (HG) conditions. (a) Cell viability was detected by the MTT assay after cells were exposed to 30 mM HG for 24, 48, and 72 h. (b) Cell viability was detected by the CCK-8 assay after cells were exposed to 5.6 mM normal glucose (NG) for 48 h. The results are expressed as the percentage of surviving cells. (c, e) Reactive oxygen species (ROS) levels in cells were detected by using a DCFH-DA probe, and the data are expressed as fluorescence intensity. (d) RMCs were treated with different concentrations of Nrf2 siRNA (30, 50, and 100 nM), and the protein levels of Nrf2 were detected by western blotting and quantified. (f) Protein levels of thioredoxin (TRX), heme oxygenase-1 (HO-1), and NAD(P)H dehydrogenase [quinone] 1 (NQO1) were detected by western blotting and quantified. Data are represented as the mean ± standard error of the mean (SEM), *n* = 3. ^#^*p* < 0.05 and ^##^*p* < 0.01 vs. the NG group; ^∗^*p* < 0.05 and ^∗∗^*p* < 0.01 vs. the HG group; ^&^*p* < 0.05 vs. the WJ-39 (10 *μ*M)+HG+control siRNA group. MTT: 3-(4,5-dimethylthiazol-2-yl)-2,5-diphenyltetrazolium bromide; CCK-8: Cell Counting kit-8; DCFH-DA: dichloro-dihydro-fluorescein diacetate.

In DN, HG promotes mesangial cell proliferation. When we cultured RMCs under HG conditions for 24, 48, and 72 h, we found higher proliferation in cells treated with HG for 48 h than in those treated for 24 h and 72 h under the same conditions. Therefore, we selected 48 h as the treatment duration to evaluate the effects of WJ-39 on RMCs. However, WJ-39 treatment did not affect the proliferation of RMCs stimulated by HG (Figure 4(a)). Hence, the effect of WJ-39 on cell viability was investigated under normal glucose (NG) conditions. Neither did WJ-39 show any significant cytotoxicity towards the RMCs (Figure 4(b)) nor did it affect cell proliferation, with or without HG. However, WJ-39 significantly reduced the excessive generation of intracellular ROS in HG-stimulated RMCs (Figure 4(c)). To determine whether the antioxidant effect of WJ-39 was dependent on the Nrf2 pathway, we transfected Nrf2 siRNA into RMCs and confirmed that 50 nM Nrf2 siRNA significantly decreased Nrf2 protein levels (Figure 4(d)). After transfection with Nrf2 siRNA, WJ-39 (10 *μ*M) did not inhibit HG-induced ROS generation in *Nrf2*-knockdown cells (Figure 4(e)). Furthermore, in cells cultured under HG conditions, the protein levels of the antioxidant enzymes HO-1, TRX, and NQO1 were significantly decreased, but this decrease was reversed by WJ-39. However, the transfection of Nrf2 siRNA eliminated this effect of WJ-39 (Figure 4(f)). These findings strongly support our hypothesis that WJ-39 exerts antioxidant effects by activating the Nrf2 pathway.

### 3.4. WJ-39 Treatment Ameliorated STZ-Induced Renal Inflammation in DN Rats

**Figure 5:**
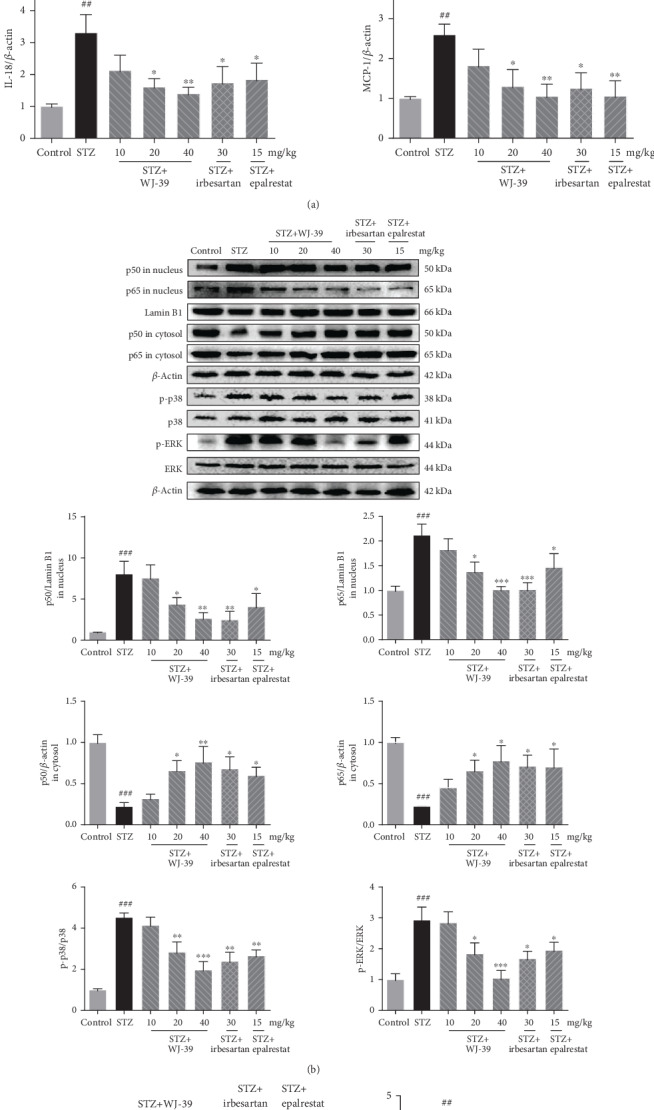
WJ-39 treatment ameliorated streptozotocin- (STZ-) induced renal inflammation in diabetic nephropathy (DN) rats. (a–c) Protein levels of interleukin-1beta (IL-1*β*), IL-6, IL-18, tumor necrosis factor-alpha (TNF-*α*), monocyte chemoattractant protein-1 (MCP-1), p50 in the nucleus and cytosol, p65 in the nucleus and cytosol, cleaved caspase-1, ASC, and NLRP3 and ratios of phosphorylated protein/total protein (p-p38/p38, p-ERK/ERK) in renal cortex tissues were detected by western blotting and quantified. Data are represented as the mean ± standard error of the mean (SEM), *n* = 6. ^##^*p* < 0.01 and ^###^*p* < 0.001 vs. the control group; ^∗^*p* < 0.05, ^∗∗^*p* < 0.01, and ^∗∗∗^*p* < 0.001 vs. the STZ group. ERK: extracellular signal-related kinase; ASC: apoptosis-associated speck-like protein containing a caspase recruitment domain; NLRP3: nucleotide-binding and oligomerization domain-like receptor family pyrin domain-containing 3.

DN is associated with renal inflammation and increased secretion of inflammatory factors, which further damage the kidneys. The levels of IL-1*β*, IL-6, IL-18, tumor necrosis factor-alpha (TNF-*α*), and monocyte chemoattractant protein-1 (MCP-1) were considerably increased in the kidneys of DN rats compared to those in normal SD rat kidneys. WJ-39 significantly suppressed the secretion of these proinflammatory cytokines (Figure 5(a)). We further investigated the effects of WJ-39 on the NF-*κ*B pathway and NLRP3 inflammasome, both of which are associated with inflammation in DN.

We observed that nuclear p65 and p50 protein levels were higher, whereas cytoplasmic p65 and p50 protein levels were lower in DN rat kidneys than in normal SD rats. Moreover, the phosphorylation levels of p38 and extracellular signal-regulated kinase (ERK), which are upstream of the NF-*κ*B pathway, were significantly increased in DN rat kidneys. Treatment with WJ-39 reversed these changes (Figure 5(b)), demonstrating that WJ-39 suppressed NF-*κ*B pathway activation in DN. Activation of the NLRP3 inflammasome leads to inflammatory injury in DN. The levels of NLRP3, adaptor protein apoptosis-associated speck-like protein containing a caspase recruitment domain (ASC), and caspase-1 were significantly higher in the kidneys of DN rats than in normal SD rats. However, this increase was dramatically inhibited by WJ-39 treatment. Epalrestat did not inhibit the NLRP3 inflammasome (Figure 5(c)). The above results indicate that WJ-39 suppressed the activation of the NF-*κ*B pathway and NLRP3 inflammasome to protect the kidneys of DN rats.

### 3.5. WJ-39 Alleviated Inflammation via the Nrf2 Pathway in RMCs Cultured under HG Conditions

**Figure 6:**
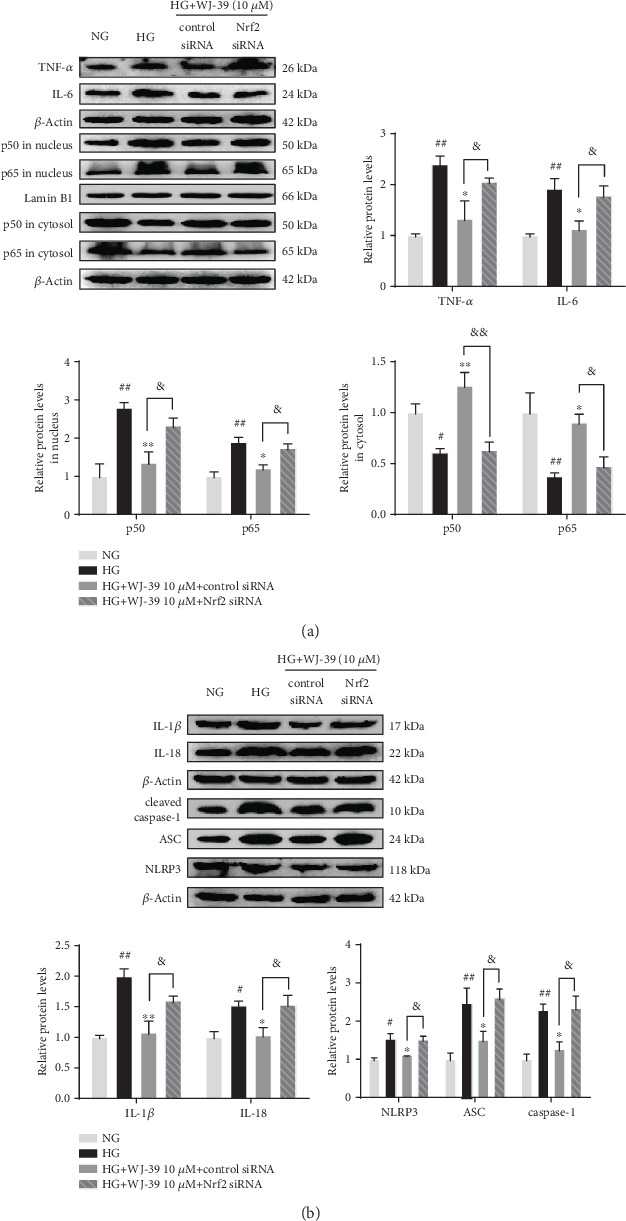
WJ-39 alleviated inflammation via the Nrf2 pathway in rat mesangial cells cultured under high glucose (HG) conditions. (a, b) Protein levels of interleukin-1beta (IL-1*β*), IL-6, IL-18, tumor necrosis factor-alpha (TNF-*α*), p50 in the nucleus and cytosol, p65 in the nucleus and cytosol, cleaved caspase-1, ASC, and NLRP3 were detected by western blotting and quantified. Data are represented as the mean ± standard error of the mean (SEM), *n* = 3. ^#^*p* < 0.05 and ^##^*p* < 0.01 vs. the normal glucose (NG) group; ^∗^*p* < 0.05 and ^∗∗^*p* < 0.01 vs. the HG group; ^&^*p* < 0.05 and ^&&^*p* < 0.01 vs. the WJ-39 10 *μ*M+HG+control siRNA group. Nrf2: nuclear factor erythroid 2-related factor 2; ASC: apoptosis-associated speck-like protein containing a caspase recruitment domain; NLRP3: nucleotide-binding and oligomerization domain-like receptor family pyrin domain-containing 3.

Evidence shows that the Nrf2 pathway inhibits inflammation and has intricate cross talk with both the NF-*κ*B pathway and NLRP3 inflammasome in DN [6, 10]. We hypothesized that WJ-39 inhibits the NF-*κ*B pathway and the NLRP3 inflammasome, which may be related to its activation of the Nrf2 pathway. To verify this hypothesis, RMCs were transfected with Nrf2 siRNA and exposed to HG. Levels of IL-6, TNF-*α*, IL-1*β*, and IL-18 were significantly higher in cells cultured under HG conditions than in cells cultured under NG conditions. In addition, the protein levels of p50 and p65 in the nucleus were significantly increased, whereas those in the cytosol were significantly decreased under HG conditions. The levels of NLRP3, ASC, and cleaved caspase-1 were also significantly increased. These changes were reversed by WJ-39; however, the effect of WJ-39 was weakened after transfection with Nrf2 siRNA (Figures 6(a) and 6(b)). Thus, the effects of WJ-39 on inflammation are dependent on activation of the Nrf2 pathway.

### 3.6. WJ-39 Treatment Prevented Renal Fibrosis by Inhibiting the TGF-*β*1/Smad Pathway in DN

**Figure 7:**
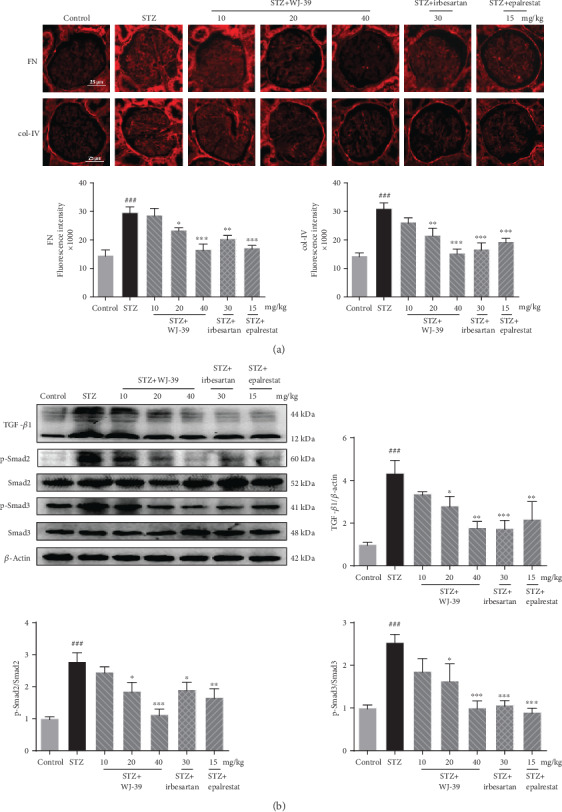
WJ-39 treatment prevented renal fibrosis by suppressing the TGF-*β*1/Smad pathway in diabetic nephropathy (DN). (a) Protein levels of fibronectin (FN) and collagen-IV (col-IV) in the glomerulus were assessed by immunofluorescence and quantified (scale bar = 25 *μ*m). (b) Protein levels of TGF-*β*1 and ratios of phosphorylated protein/total protein (p-Smad2/Smad2, p-Smad3/Smad3) in renal cortex tissues were detected by western blotting and quantified. Data are represented as the mean ± standard error of the mean (SEM), *n* = 6. ^###^*p* < 0.001 vs. the control group; ^∗^*p* < 0.05, ^∗∗^*p* < 0.01, and ^∗∗∗^*p* < 0.001 vs. the streptozotocin (STZ) group. TGF-*β*1: transforming growth factor-beta1.

WJ-39 prevented renal lesions in DN by reducing oxidative stress and inflammation, both of which ultimately contribute to renal fibrosis. Levels of fibronectin (FN) and collagen-IV (col-IV), the main proteins of ECM, were measured. The expression of FN and col-IV was significantly higher in the glomeruli of DN rat renal tissues than in normal SD rats; WJ-39 treatment markedly reduced their protein levels (Figure 7(a)). Levels of TGF-*β*1 protein and the phosphorylation levels of Smad2 and Smad3 were significantly elevated in DN rat kidneys compared to those in normal SD rats. However, WJ-39 markedly suppressed the activation of the TGF-*β*1/Smad pathway (Figure 7(b)), indicating that WJ-39 mitigated the aggregation of ECM proteins in the glomeruli of DN rats by blocking the TGF-*β*1/Smad pathway.

## 4. Discussion

The polyol pathway has a crucial role in the progression of DN, and inhibiting AR may be a potential therapeutic strategy to prevent and treat DN [25]. In this study, WJ-39, a novel compound, effectively inhibited the activity of AR in renal tissues of STZ-induced DN rats. We found that, unlike epalrestat, WJ-39 did not decrease AR protein expression. Differences in the effects of ARIs on AR expression have been reported. Although epalrestat reduces the expression of AR, fidarestat, sorbinil, and zopolrestat do not; however, they all inhibit AR activity [26–28]. We speculate that epalrestat might affect the mRNA of AR or cause the degradation of AR protein, whereas WJ-39 did not; the underlying mechanism needs further study. We also found that WJ-39 markedly increased Ccr and reduced ACR, mesangial matrix index, and percentage of fibrosis in renal tissues of DN rats, indicating that WJ-39 ameliorated renal dysfunction and fibrosis in DN rats. Therefore, we hypothesized that the renoprotective effect of WJ-39 in DN might be attributed to its regulation of the polyol pathway.

The polyol pathway includes two-step enzymatic reactions. First, glucose is transformed to sorbitol by AR, increasing the turnover of reduced nicotinamide-adenine dinucleotide phosphate (NADPH), a cytosolic cofactor of AR. Then, the oxidized form, NADP^+^, acts as a cofactor for glutathione reductase, directly leading to a decrease in the levels of a critical antioxidant, GSH [29]. Second, sorbitol dehydrogenase converts sorbitol and the cofactor NAD^+^ into fructose and NADH, respectively [30]. NADH participates as an electron donor to activate mitochondrial metabolism with a subsequent increase in superoxide production [17, 31–33]. Both steps of the polyol pathway lead to intracellular oxidative stress. Inhibition of AR activity suppresses the occurrence of renal oxidative stress and ROS production [26, 34]. Consistent with this finding, we found that WJ-39 significantly increased the ratios of GSH/GSSG and NAD^+^/NADH and decreased the concentration of MDA, a biomarker that indicates the extent of cell damage caused by oxidative stress [35]. Furthermore, WJ-39 significantly reduced ROS generation induced by HG in RMCs. Nrf2 is an important defense against oxidative stress. Under conditions of oxidative stress, Nrf2 translocates to the nucleus and activates the antioxidant response element, promoting the expression and activity of antioxidants, such as HO-1 and SOD [36]. However, chronic oxidative stress or hyperglycemic stimulation abolishes the antioxidant capacity of Nrf2 [37]. Inhibition of AR activated the Nrf2 pathway in the STZ-induced diabetic mice kidneys [38]. In our current study, nuclear Nrf2 levels decreased in the STZ-induced DN rat kidneys, while the expression or activity of the downstream antioxidant enzymes, HO-1, NQO1, TRX, SOD, and CAT also decreased. These effects were reversed by WJ-39 treatment. WJ-39 also increased the phosphorylation of PI3K and AKT, both important proteins in a kinase pathway that activates Nrf2 [39]. To further verify whether the antioxidant role of WJ-39 in DN is linked to the Nrf2 pathway, *Nrf2* was knocked down in RMCs by Nrf2 siRNA. Knockdown of *Nrf2* suppressed the increase of antioxidant enzyme levels after WJ-39 treatment. These findings demonstrate that WJ-39 ameliorates oxidative stress in DN and is associated with the activation of the Nrf2 signaling pathway.

Several lines of evidence indicate that inflammation is critically involved in the progression of DN [40, 41]. The proinflammatory transcription factor, NF-*κ*B, is thought to be the main cause of renal inflammatory injury, regulating the expression of genes that are involved in the inflammatory response [42]. Inactive NF-*κ*B normally exists in the cytoplasm. When responding to stimulation, NF-*κ*B translocates into the nucleus and triggers the gene expression of cytokines and chemokines such as IL-6, TNF-*α*, and MCP-1 [43, 44]. The NF-*κ*B pathway is activated and leads to inflammation and fibrosis-related damage in STZ-induced DN model animals and HG-induced mesangial cells [45]. Accumulating evidence also suggests that ERK and p38 MAPK function as signaling intermediates in DN. Resultant hyperphosphorylation of p38 MAPK in diabetes, which is regulated by ROS and HG levels, triggers NF-*κ*B signaling in renal injury [32, 46–49]. In the present study, the results show that WJ-39 reduced the protein levels of p50 and p65 in the nucleus and blocked the NF-*κ*B pathway to significantly attenuate STZ-induced secretion of IL-6 and TNF-*α* in renal tissues. WJ-39 also abrogated the increase in phosphorylation of p38/ERK in DN rat kidneys. These results illustrate that WJ-39 inhibits the NF-*κ*B pathway. Many researchers have also proposed that there is cross talk between NF-*κ*B and Nrf2. The Nrf2 pathway reduces the phosphorylation of NF-*κ*B inhibitor alpha (IkB*α*), thereby inhibiting NF-*κ*B activity [6]. NF-*κ*B activity is also suppressed by HO-1 through inhibition of IkB degradation [50]. *HO-1* knockout mice show increased NF-*κ*B activity and secretion of inflammatory factors, such as MCP-1, a macrophage/monocyte chemokine [51–53]. In this study, transfection with Nrf2 siRNA inhibited the effects of WJ-39 on the NF-*κ*B pathway. Therefore, it can be concluded that the effect of WJ-39 on the NF-*κ*B pathway relies on the activation of the Nrf2 pathway.

Another crucial pathway that promotes renal inflammation in DN is the NLRP3 inflammasome. The NLRP3 inflammasome is an intracellular multiprotein complex consisting of NLRP3, ASC, and caspase-1. The NLRP3 inflammasome is activated by oxidative stress and hyperglycemia in DN [54–56]. NF-*κ*B is also involved in the regulation of *NLRP3* gene expression [57]. When endogenous danger signals are detected, NLRP3 binds to ASC, recruits pro-caspase-1 to assemble into the inflammasome, and then activates caspase-1, which induces IL-1*β* and IL-18 secretion, leading to sustained inflammation [54]. Some studies have found that the NLRP3 inflammasome is activated in the glomeruli of DN patients and STZ-induced DN models and in glomerular mesangial cells cultured under HG stress, and that this activation is accompanied by elevated levels of IL-1*β* and IL-18 [56, 58, 59]. Therefore, suppression of NLRP3 inflammasome activation alleviates renal injury in DN. Pal et al. reported that AR activated the NLRP3 inflammasome in HG-induced THP1 monocytes and in the heart and aorta of STZ-induced diabetic mice [60]. Further, the levels of NLRP3 inflammasome components were reduced by inhibition of AR in STZ-induced diabetic mouse hearts [60], but the role of ARIs on NLRP3 inflammasome in the kidneys has not been studied. In this study, we observed that treatment with WJ-39 markedly reduced the levels of NLRP3 components in renal cortex tissues of DN rats and HG-induced RMCs. We hypothesized that WJ-39 inhibits the NLRP3 inflammasome by suppressing AR activity, but the mechanism is unclear. Shahzad et al. found that stabilization of endogenous Nrf2 protected the kidneys via inhibition of the NLRP3 inflammasome, and this inhibition was abolished in the kidneys of diabetic *Nrf2*^−/−^mice [61]. Consistent with this finding, our results also show the abolition of the inhibitory effect of WJ-39 on the NLRP3 inflammasome after Nrf2 siRNA transfection in HG-induced RMCs. Thus, it can be concluded from the above findings that the effect of WJ-39 on the NLRP3 inflammasome through the inhibition of AR activity is associated with the activation of the Nrf2 pathway.

Renal fibrosis is characterized by the excessive deposition of ECM proteins such as col-IV and FN. Such aberrant ECM accumulation leads to glomerulosclerosis, culminating in the progression of renal dysfunction [62, 63]. TGF-*β*1 is a major driver in kidney fibrosis and can be stimulated by a variety of factors including HG, oxidative stress, and cytokines such as IL-1*β* [63]. Active TGF-*β*1 stimulates ECM synthesis by activating Smad pathways [64, 65]. Hence, blocking TGF-*β*1 activity may have positive effects in the intervention of DN. In our study, we observed an increase in ECM protein levels in the glomeruli of STZ-induced DN rats, which was partially reversed by WJ-39 treatment. According to some reports, ARIs prevent HG-induced increase in TGF-*β*1 in mesangial cells [66, 67]. Thus, the antifibrotic effect of WJ-39 might be related to its blockade of TGF-*β*1/Smad pathway through the inhibition of AR activity; however, the underlying molecular mechanisms linking TGF-*β*1 and AR need further study. In this study, we found the protective effect of WJ-39 on glomerular mesangial cells; however, various types of renal cells are injured and are involved in the progression of DN, such as podocytes and renal tubular epithelial cells [68, 69]. We will explore the effect and mechanism of WJ-39 on other renal cells in a future study.

## 5. Conclusions

**Figure 8:**
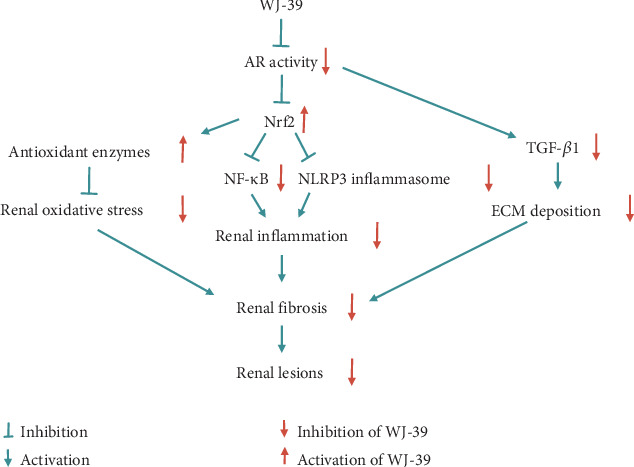
The hypothetical molecular mechanism of WJ-39 in diabetic nephropathy. AR: aldose reductase; Nrf2: nuclear factor erythroid 2-related factor 2; NF-*κ*B: nuclear factor-kappa B; NLRP3: nucleotide-binding and oligomerization domain-like receptor family pyrin domain-containing 3; TGF-*β*1: transforming growth factor-*β*1; ECM: extracellular matrix.

The molecular mechanisms underlying the occurrence and progression of DN are complex. An effective strategy is urgently needed to prevent the progression of DN. WJ-39 ameliorated renal injury by inhibiting oxidative stress, inflammation, and fibrosis in DN rats. After transfection with Nrf2 siRNA, the inhibitory effects of WJ-39 on ROS production, NF-*κ*B pathway, and the NLRP3 inflammasome were abolished in RMCs cultured under HG conditions. Therefore, the effects of WJ-39 on oxidative stress and inflammation were associated with the activation of Nrf2 signaling. Our present findings indicate that WJ-39 inhibited the activity of AR and effectively ameliorated renal inflammation, oxidative stress, and fibrosis injury, all of which could comprise an effective strategy for the treatment of DN (Figure 8).

## Figures and Tables

**Table 1:** Primary antibody information for western blotting.

Primary antibodies	Catalog number, supplier
Anti-thioredoxin (TRX)	14999-1-AP, Proteintech, Wuhan, China
Anti-NAD(P)H dehydrogenase [quinone] 1 (NQO1)	ab80588, Abcam, Cambridge, UK
Anti-HO-1	ab68477, Abcam, Cambridge, UK
Anti-AR	15439-1-AP, Proteintech, Wuhan, China
Anti-phosphatidylinositol-3-kinase (PI3K)	#4257, Cell Signaling Technology, Danvers, MA, USA
Anti-p-PI3K	SAB4504314, Sigma-Aldrich, St. Luis, MO, USA
Anti-AKT	sc-81434, Santa Cruz, Dallas, Texas, USA
Anti-p-AKT	sc-514032, Santa Cruz, Dallas, Texas, USA
Anti-Nrf2	ab137550, Abcam, Cambridge, UK
Anti-TGF-*β*1	ab179695, Abcam, Cambridge, UK
Anti-IL-6	21865-1-AP, Proteintech, Wuhan, China
Anti-p50	ab32360, Abcam, Cambridge, UK
Anti-p65	ab16502, Abcam, Cambridge, UK
Anti-Lamin B1	12987-1-AP, Proteintech, Wuhan, China
Anti-ERK	16443-1-AP, Proteintech, Wuhan, China
Anti-p-ERK1/2	ab76299, Abcam, Cambridge, UK
Anti-p38	14064-1-AP, Proteintech, Wuhan, China
Anti-p-p38 (T180+Y182)	ab4822, Abcam, Cambridge, UK
Anti-NLRP3	NBP2-12446, Novus, Littleton, CO, USA
Anti-Smad3	25494-1-AP, Proteintech, Wuhan, China
Anti-p-Smad2 (ser465/ser467)	#18338, Cell Signaling Technology, Danvers, MA, USA
Anti-p-Smad3 (ser423/ser425)	#9520, Cell Signaling Technology, Danvers, MA, USA
Anti-caspase-1	ab179515, Abcam, Cambridge, UK
Anti-monocyte chemoattractant protein-1 (MCP-1)	66272-1-lg, Proteintech, Wuhan, China
Anti-Smad2	12570-1-AP, Proteintech, Wuhan, China
Anti-apoptosis-associated speck-like protein containing a caspase recruitment domain (ASC)	sc-514414, Santa Cruz, Dallas, Texas, USA
Anti-IL-1*β*	WL00891, Wanleibio, Shenyang, China
Anti-IL-18	10663-1-AP, Proteintech, Wuhan, China
Anti-tumor necrosis factor-*α* (TNF-*α*)	60291-1-Ig, Proteintech, Wuhan, China
Anti-*β*-actin	sc-47778, Santa Cruz, Dallas, Texas, USA

## Data Availability

The data used to support the findings of this study are available from the corresponding author upon request.
